# Association mapping of blood pressure levels in a longitudinal framework using binomial regression

**DOI:** 10.1186/1753-6561-8-S1-S74

**Published:** 2014-06-17

**Authors:** Arunabha Majumdar, Indranil Mukhopadhyay, Saurabh Ghosh

**Affiliations:** 1 Human Genetics Unit, Indian Statistical Institute, 203 B.T. Road, Kolkata 700108, India

## Abstract

Heritable quantitative characters underline complex genetic traits. However, a single quantitative phenotype may not be a suitably good surrogate for a clinical end point trait.

It may be more optimal to use a multivariate phenotype vector correlated with the end point trait to carry out an association analysis. Existing methods, such as variance components and principal components, suffer from inherent limitations, such as lack of robustness or difficulty in biological interpretation of association findings. In an effort to circumvent these limitations, we propose a novel regression approach based on a conditional binomial model to detect association between a single-nucleotide polymorphism and a multivariate phenotype vector. We use our proposed method to analyze data on systolic and diastolic blood pressure levels provided in Genetic Analysis Workshop 18. We find that the bivariate analysis of the two phenotypes yields more promising results in terms of lower *p*-values compared to univariate analyses.

## Background

Most clinical end point traits are governed by a set of quantitative precursors and a single precursor is unlikely to explain a significant amount of the variation in the end point trait. Thus, it may be a prudent strategy to analyze a multivariate vector of phenotypes for association mapping of a clinical end point trait. The major statistical challenge in the analyses of multivariate phenotypes lies in the modeling of the vector of phenotypes. Univariate analyses of the constituent phenotypes may lead to the statistical problem of multiple comparisons [1]. Likelihood-based methods, such as variance components [2,3], are susceptible to the choice of the joint probability distribution of the components of the vector. An alternative approach that circumvents the problem of modeling the multivariate phenotype is to obtain a reduced univariate phenotype using principal components [4]. However, association results based on principal components may be difficult to interpret biologically. We propose a novel binomial regression approach that models the likelihood of the number of copies of the minor allele at a single-nucleotide polymorphism (SNP) conditional on the vector of multivariate phenotype. We apply our proposed method to analyze systolic and diastolic blood pressure levels on unrelated individuals using longitudinal data over four time points provided in Genetic Analysis Workshop 18 (GAW18). We compare the association results based on a bivariate analysis of the two phenotypes with those based on univariate analyses.

### Data description

For our analyses, we use data on systolic blood pressure (SBP) levels and diastolic blood pressure (DBP) levels on 157 unrelated individuals along with their genotypes at all the available 456,752 variant sites distributed over 11 autosomal chromosomes in the genome-wide association studies data provided in GAW18. In addition to age, we use smoking status and medication indicator (both defined as binary variables) at each time point of examination as covariates, as these factors could be potential confounders in the association analyses. Both the SBP and the DBP levels are adjusted for these covariates for each time point and the tests for association are performed on the adjusted phenotypes. The adjustment and the association analyses are based on 139 individuals, as data on all the covariates are not available for the remaining individuals. The percentages of individuals with missing phenotype data at the different time points are 8%, 35%, 35%, and 75%, respectively. On tabulating the percentages of individuals with phenotype data missing at multiple time points, we do not observe any pattern in the missing data. For our association analyses, we impute the missing data using a procedure outlined in the following section.

## Methods

### Statistical methodology

#### *Imputation of missing phenotype values and covariate adjustment*

The assumption of multivariate normality provides a computationally elegant framework for the expectation maximization algorithm [5] to estimate parameters when data are missing. Blood pressure levels have traditionally been believed to follow a lognormal distribution. While the Kolmogorov-Smirnov test did not show any significant departure from normality for the systolic and diastolic blood pressure levels at any of the time points, some of the *p-*values are very close to the threshold of 0.05. We thus use a logarithmic transformation on each of the phenotypes to induce normality. Suppose the vector of log-transformed values of any of the two phenotypes at the 4 time points is represented as ***X ***= (*X*_1_, *X*_2_, *X*_3_, *X*_4_). If ***Y ***denotes the vector comprising those components of ***X ***that are missing and ***Z ***comprising the components that are available for an individual, ***Y ***is estimated via an expectation maximization algorithm as the expectation of ***Y ***conditioned on ***Z ***and is given by **μ_Y_-∑_YZ_∑_ZZ_^-1^(Z-μ_Z_), **where, **μ_Y _**and **μ_Z _**are the mean vectors of ***Y ***and ***Z***, respectively; ∑_YZ_ is the matrix of covariance between ***Y ***and ***Z***, while **∑_ZZ _**is the dispersion matrix of ***Z***. At every time point, we perform a linear regression of the log-transformed values of each of the two phenotypes (available as well as imputed) on age, smoking status, and medication indicator based only on those observations for which data were available on all the covariates and thereby obtain the residuals of each of the regressions.

#### *Test for association using binomial regression*

The phenotypes for our association analyses are the adjusted SBP and DBP levels at each time point obtained using the algorithm described in the preceding section. We propose a binomial regression framework to test for association of a SNP with a multivariate phenotype. Suppose ***X ***= (*X*_1_, *X*_2_, *X*_3_,..., *X*_k_) denotes a vector of *k *phenotypes and ***N ***denotes the number of copies of the minor allele (0, 1, or 2) at a SNP. We model the conditional distribution of ***N ***given ***X ***as binomial with parameters 2 and ***p***(***X***) where, ***p***(***X***) is a logistic link function given by:

$$\frac{\text{exp}\left(\beta_{0}+\sum_{i=1}^{k}\beta_{i}X_{i}\right)}{1+\text{exp}\left(\beta_{0}+\sum_{i=1}^{k}\beta_{i}X_{i}\right)}$$

The test for association is equivalent to testing *H*_0_: β_1 _=β_2_= ... =β_k _= 0 versus *H*_1_: not *H*_0 _and the log-likelihood ratio test statistic is distributed as chi-squares with *k *degrees of freedom under the null hypothesis. We compare the relative performances of 5 phenotype vectors in detecting association: (a) *T*_1_: the adjusted SBP levels at the 4 time points; (b) *T*_2_: the adjusted DBP levels at the 4 time points; (c) *T*_3_: the adjusted SBP levels summarized by the first 2 principal components across the four time points; (d) *T*_4_: the adjusted DBP levels summarized by the first two principal components across the four time points; and (e) *T*_5_: the adjusted SBP as well as the adjusted DBP levels summarized by a bivariate phenotype comprising the first 2 principal components corresponding to each of the phenotypes across the four time points. The above choice of the principal components is motivated by the fact that approximately 75% of the variation in each of the two phenotypes is explained by the corresponding first two principal components. Because the sample size is not very large, we consider only common variants for our association analyses and select only those SNPs with a minor allele frequency of at least 0.01. We also exclude those SNPs that provide significant evidence of departure from Hardy-Weinberg equilibrium (based on the Bonferroni corrected threshold at the genome-wide level). Thus, the tests for association based on the proposed binomial regression model are carried out on 426,193 SNPs. To correct for multiple testing, we use the false discovery rate (FDR) procedure [6] with an overall rate of 0.05.

## Results

Among the five phenotype vectors considered, the one comprising the first two principal components of both SBP and DBP levels (*T*_5_) provides the most promising association findings. Table 1 presents the five most significant association findings corresponding to each choice of phenotype vector. The only SNP that exhibits significant evidence of association at the genome-wide level is *rs12634258 *on chromosome 3 (adjusted FDR of 0.047) with *T*_5_. We observe that this SNP also ranks among the top five SNPs in terms of lowest unadjusted *p-*values when *T*_3 _(the first two principal components of the SBP levels at the four time points) is analyzed.

**Table 1:** Five most significant association findings using the different phenotype vectors

*T*_1_	*T*_2_	*T*_3_	*T*_4_	*T*_5_
rs630685 (3 × 10^−6^, 1q42.1)	rs7928749 (1 × 10^−6^, 11q22.2)	rs11233253 (4.9 × 10^−6^, 11q14.1)	rs4239000 (9.3 × 10^−6^, 17q25)	rs12634258 (1 × 10^−7^, 3p14.2)
rs1789956 (3.5 × 10^−6^, 21q22.3)	rs1129174 (5.9 × 10^−6^, 3p25)	rs11212913 (7.7 × 10^−6^, 11q22.3)	rs6590606 (2 × 10^−5^, 11q25)	rs12153302 (1.1 × 10^−6^, 5q13.2)
rs3829088 (3.8 × 10^−6^, 9p21)	rs6791435 (6.4 × 10^−6^, 3p25.1)	rs9632874 (9.4 × 10^−6^, 9p22.3)	rs7117235 (2.1 × 10^−5^, 11q25)	rs12652232 (3.3 × 10^−6^, 5q35.3)
rs12591997 (4.1 × 10^−6^, 15q21.2)	rs4685160 (9.5 × 10^−6^, 3p25.1)	rs16960959 (1.1 × 10^−5^, 17p11.2)	rs1870301 (2.9 × 10^−5^, 15q26.2)	rs7541416 (3.9 × 10^−6^, 1p36.1)
rs12915086 (6.2 × 10^−6^, 15q26)	rs894177 (1.6 × 10^−5^, 3q24)	rs12634258 (1.2 × 10^−5^, 3p14.2)	rs12153302 (3.9 × 10^−5^, 5q13.2)	rs7644778 (4.6 × 10^−6^, 3q28)

The figures in parentheses represent the unadjusted *p-*values and chromosomal locations, respectively.

## Conclusions

We have developed a binomial regression model that can incorporate multiple phenotypes for association analysis of the multivariate phenotype vector. The method has several advantages: it neither involves any modeling of the correlation structure within the components of the multivariate phenotype as required in likelihood-based approaches nor does it compromise on the biological interpretation by transforming the multivariate phenotype to principal components.

The SNP *rs12634258 *that exhibits the most promising evidence of association with multiple definitions of the phenotype vector is located in the intergenic region between the genes *PTPRG *(receptor-type tyrosine-protein phosphatase gamma) and *FHIT *(fragile histidine triad) on 3p14.2. The RNA expression in *PTPRG *has been reported to be upregulated for arrhythmogenic right ventricular cardiomyopathy in humans [7]. On the other hand, the RNA expression of *FHIT *is downregulated in mouse for low blood pressure [8]. Although we obtain only one association finding that satisfies the genome-wide FDR threshold of 0.05, it is likely that the sample size is grossly inadequate to detect associations for a complex phenotype.

While constructing the phenotype vectors, we had considered the first two principal components of the blood pressure levels across the four time points. As mentioned earlier, these two principal components explain approximately 75% of the total variation of blood pressure levels across the time points. Even though the first principal component explains approximately 55% of the variation, the first 3 principal components explain approximately 90% of the variation. Although for brevity we do not present the association findings for the different choices of the principal components, we find that the SNP *rs7644778 *ranks among the top two association findings with the first principal component of each of SBP and DBP levels across the four time points, albeit not significant at the genome-wide level. We note that this SNP also exhibits evidence of association with first two principal components of each of the two phenotypes. The SNP is located in the gene *TPRG1 *(tumor protein p63 regulated 1). The RNA expression of this gene has been reported to be upregulated for cardiomyopathy in humans. On the other hand, the five most significant findings with the first two principal components are identical to those with the first three principal components, but none of them is significant at the genome-wide level. This may be explained by the fact that the test with 3 principal components for each of the two phenotypes has two additional degrees of freedom under the null hypothesis and may suffer from loss in power.

Among the five phenotype vectors (*T*_1_, *T*_2_, *T*_3_, *T*_4 _and *T*_5_), we find that *T*_5 _(ie, the phenotype that considers both SBP as well as DBP levels simultaneously) provides the most significant association findings (in terms of *p-*value). Even though the empirical findings seem to suggest that association analyses based on a multivariate phenotype comprising traits modulated by common genetic variants are likely to be more powerful than univariate analyses of the constituent traits of the multivariate phenotype vector, such a hypothesis can only be validated using extensive simulations. We have performed some independent simulations on 500 unrelated individuals under a few pleiotropic bivariate phenotype models: (a) the constituent traits have a bivariate normal distribution, (b) both are distributed as chi-squares, and (c) 1 trait has a normal distribution, while the other is binary. Our preliminary results based on 1000 replications indicate that our proposed method maintains the correct false-positive rates under all the phenotype models. We also find that simultaneous analyses of both the phenotypes as covariates consistently yield more power compared to analyzing each univariate trait using the binomial regression. On the other hand, the power of the proposed method based on both phenotypes is higher, similar, or marginally lower compared to that based on the first principal component, depending on whether the correlation between the traits is low (less than 0.25), medium (between 0.25 and 0.5) or high (greater than 0.5). The above phenomenon was observed irrespective of the bivariate phenotype model considered. When the gene effects are in different directions for the two phenotypes (the minor homozygous genotype corresponds to high values of 1 phenotype, but low values of the other phenotype), the first principal component performs very poorly and may yield lower power compared to analyzing univariate phenotypes. However, further simulations based on higher dimensional phenotype vectors need to carried out to obtain greater insights into the relative advantages of the proposed method using the multivariate phenotype vector over data reduction approaches such as using a subset of the principal components of the phenotype vector.

## Competing interests

The authors declare that they have no competing interests.

## Authors' contributions

SG and AM developed the proposed method. AM and IM wrote the computer codes and performed the data analyses. AM participated in the compilation and interpretation of the results. All authors read and approved the final manuscript.
